# RECURRENCE PATTERN IN SQUAMOUS CELL CARCINOMA OF SKIN OF LOWER EXTREMITIES AND ABDOMINAL WALL (KANGRI CANCER) IN KASHMIR VALLEY OF INDIAN SUBCONTINENT: IMPACT OF VARIOUS TREATMENT MODALITIES

**DOI:** 10.4103/0019-5154.57610

**Published:** 2009

**Authors:** Mohmad Ashraf Teli, N A Khan, M Ashraf Darzi, Meenu Gupta, A Tufail

**Affiliations:** *From the Department of Radiation Oncology, SKIMS, Soura, Srinagar - 190 011, Kashmir, India.*; 1 *From the Department of Plastic & Reconstructive Surgery, SKIMS, Soura, Srinagar - 190 011, Kashmir, India.*

**Keywords:** *Erythema ab igne*, *Kangri cancer*, *lymph nodal irradiation*, *squamous cell carcinoma*

## Abstract

**Background:**

The spectrum of skin cancer in Kashmir valley is drastically different from the rest of the country. Maxwell was the first to report skin cancer of lower extremities in Kashmiri population, developing on/over erythema ab igne, and attributed it to the use/or exposure of Kangri. These tumors have an aggressive biological behavior with a substantial risk of loco-regional metastasis in 30-50% cases. Because of unique geographical distribution of Kangri cancer, there is dearth of literature regarding the natural history, loco-regional and distant metastatic pattern and treatment recommendations in these tumors.

**Aims:**

To study the metastatic pattern of these skin tumors and to assess the impact of various treatment modalities and use of prophylactic nodal treatment in this clinical entity.

**Methods:**

The retrospective study (study period 1993-2005) included 266 patients of squamous cell carcinoma of skin of lower extremities and abdominal wall. Two hundred and forty-four cases with a follow-up of 2-7 years were included for final analysis with stress on loco-regional relapse pattern and methods of treatment evolved and used at our institute from time to time. Statistical analysis was done using yates corrected Chi-square test and odds ratio analysis.

**Results:**

Our results favor the use of post operative radiotherapy to primary and prophylactic treatment of regional nodes on the lines of head and neck tumors in these cases.

**Conclusion:**

Post operative radiotherapy significantly decreases the loco-regional recurrences and a trial of prophylactic nodal irradiation is justified in a selected group of such patients.

## Introduction

**Figure 1:**
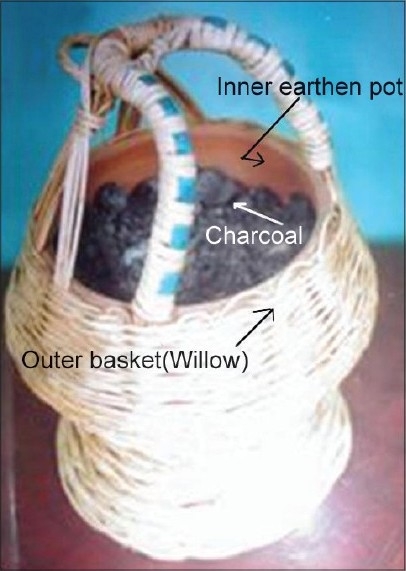
Components of Kangri

**Figure 2:**
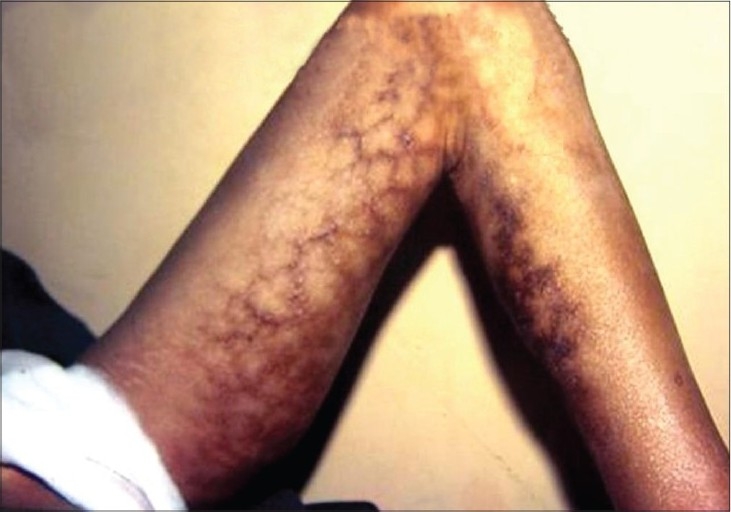
Erythema abigne on medial aspect of thigh and leg

**Figure 3:**
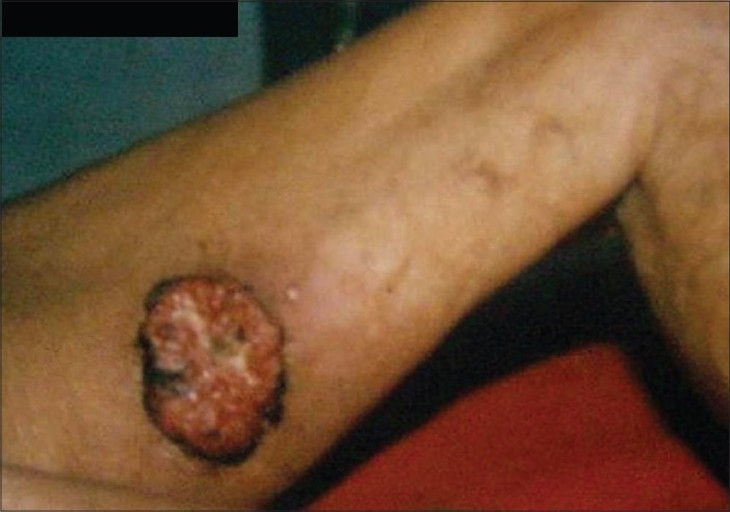
A T3 Kangri cancer

**Figure 4:**
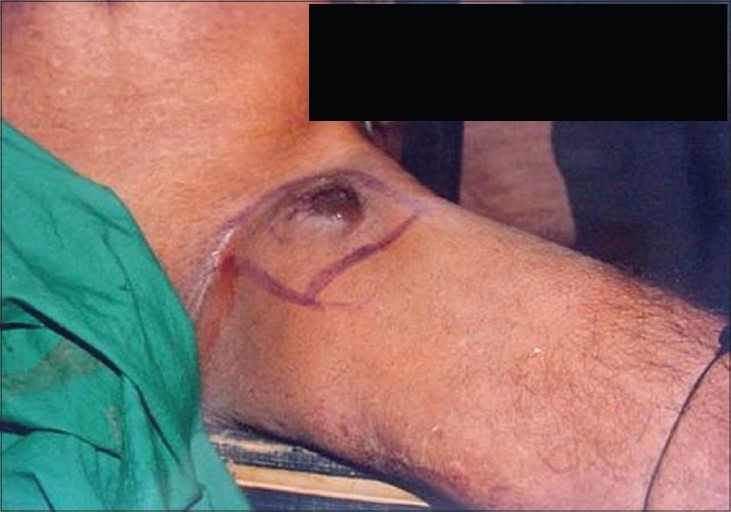
Post operative nodal recurrence

**Figure 5:**
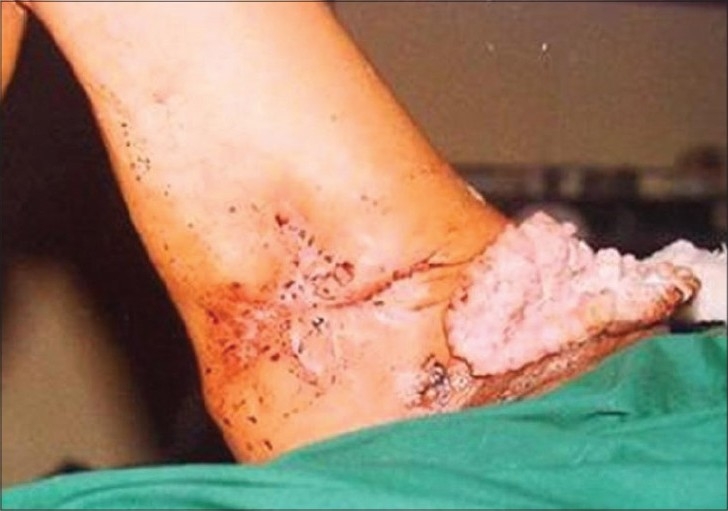
Post operative fungating nodal recurrence

Non-melanoma skin cancer is the most common form of skin cancer with basal cell carcinoma outnumbering the squamous cell carcinoma in majority of the geographical regions of world.[1,2] The most obvious and significant cause is exposure to sunlight (ultraviolet radiation) and as such, it usually occurs in sun exposed anatomical sites[3] However, various studies from India consistently report squamous cell carcinoma as the most prevalent skin malignancy.[4,5] Clinical spectrum of skin cancer in Kashmir valley is different from the rest of the country. Maxwell, in 1879, was the first to report skin cancer of the lower extremities from Kashmir valley and attributed it to the use/exposure of Kangri [Figure 1] – an indigenous fire pot used and tucked in between the thighs to generate warmth during the winter months.[6] Kangri cancer usually starts as a papular growth on/over pre existing thermal keratotic lesions called erythema ab igne [Figure 2]. With time these lesions usually ulcerate and grow exponentially [Figure 3]. These tumors have an aggressive biological behavior with a substantial risk of loco-regional metastasis in 20-50% cases.[7–9] [Figures 4 and 5] Because of its unique geographical distribution, there is dearth of literature regarding the natural history and the spectrum of loco-regional and distant metastatic pattern in these tumors. The present study was undertaken to study the metastatic pattern in these tumors and to assess the impact of various treatment modalities used in this clinical entity.

## Materials and Methods

From 1993 to 2005, a total of 537 patients of skin cancers were registered in the departmental cancer registry. Two hundred and sixty six (49.5%) cases with histopathological documentation and location in the lower extremities and abdominal wall were enrolled for the present study. The demographic and various clinical parameters were recorded in detail [Table 1]. Pre-treatment evaluation consisted of detailed history with stress on the use of Kangri, detailed physical examination, CBC, chest radiography and an abdominal and pelvic ultrasound. A high energy ultrasound/CT scan was done in patients with abdominal wall tumors to estimate the depth of tumor and to assess the status of underlying structures. The staging was based on clinical and pathological details and on the guidelines of International Union Against Cancer. The staging was fortified by detailed surgical and pathological details. Surgical treatment in the form of wide resection/excision was performed in all patients. Radiotherapy was the main modality of treatment in post operative setting especially in patients with positive resection lines/peri-neural/peri-vascular infiltration and loco-regional recurrent disease after surgery. In postoperative setting, a dose of 50-55 Gy was used to treat the primary site and 45 Gy (prophylactic) to regional (1^st^. echelon) nodes by a direct field; using a cobalt unit with bolus wherever needed using conventional fractionation schedule. A dose of 55-65 Gy was used to treat primary lesion in 22 cases and 70 cases with inguino-femoral nodal disease. Follow-up ranged from 2 to 7 years. The target volume included the scar area with a generous margin and treatment was delivered on a tele-cobalt unit using bolus in all cases. The abdominal wall tumors were treated by two tangential or oblique portals after ascertaining the depth by a high energy ultrasound and a CT-scan. Plato treatment planning system (Nucletron make) was used to verify the dose distribution. The inguino-femoral nodes were treated by a direct anterior field. The overall protocol design for post-operative radiotherapy in general and for prophylactic nodal irradiation is shown in Table 2.

**Table 1:** Distribution of cases according to various demographic and clinical parameters (n = 266)

Parameter	No	%
Sex		
Males	156	58.64
Females	110	41.36
Habitat		
Rural	183	68.80
Urban	83	31.20
Age		
Less than 40 years	12	04.51
41-50 years	43	16/.16
51-60 years	78	29.32
61-70 years	99	37.21
More than 70 years	34	12.78
Site of involvement		
Thighs (total)	191/266	71.80
Right	102/191	53.40
Left	89/191	46.60
Anterior abdominal wall	59/266	22.18
Legs (total)	15/266	05.63
Right	08/15	53.33
Left	07/15	46.67
Feet	01/266	00.37
Histopathological differentiation		
Well differentiated	192	72.18
Moderately differentiated	16	06.01
Poorly differentiated	03	01.12
Not specified	55	20.67

**Table 2:** Overall and prophylactic nodal irradiation (treatment design)

T1 lesion – Surgery	T2 lesions – Surgery	T3 lesions – Surgery
a) R0 status – Close follow-up	a) R0 status – Prophylactic nodal RT alone	Post OP. RT to local site (55 Gy) to all + Prophylactic Inguino-femoral nodal RT to all
b) R1 status – LVI/peri-neural invasion – doubtful marginal status – Post OP. RT to local site only (50 Gy)	b) R1 status – LVI – peri-neural invasion doubtful marginal status	
	– Post OP. RT to local site (55 Gy)	
	+ Prophylactic IFN RT	

Dose of RT local site: 50-55 Gy/ 5-5.5 weeks; R0 = Negative resection line status; R1 = Positive resection line status, RT = Radiotherapy; LVI = Lymph vascular invasion; IFN RT = Inguino-femoral nodal radiotherapy. Note: Prophylactic-inguino-femoral nodes = 45 Gy/4 weeks - given to patients with N0/Nx status only.

## Results

Between 1993 and 2005, 12848 patients were registered in the Departmental cancer registry. Of these 537 (04.17%) cases had skin cancer. Two hundred and sixty six patients with history of chronic use/exposure of Kangri and having Kangri induced thermal keratotic patches in the form of erythema ab igne around the primary lesion were labeled to have Kangri cancer. There were 156 males and 110 female patients. Majority of patients were in the age group of 50-70 years. The primary site of the tumor was antero-medial aspect of thigh in 191 cases, anterior abdominal wall in 59 patients and legs/feet in 16 patients. One hundred and seventy four patients had stage II disease; 62 cases stage III while stage I was seen in 30 patients only. Of the 266 cases of squamous cell carcinoma, 192 had well differentiated sub-type [Table 1]. Two hundred and forty four cases were subjected to surgery of which 83 having positive resection lines/lymphovascular/peri-neural invasion or doubtful marginal status received post operative radiotherapy to local site. These patients received post operative radiotherapy to primary site (Dose ranging from 50-55 Gy/5-5.5 weeks). Twenty two patients with locally advanced disease and not willing for surgery were treated with radiotherapy alone (excluded from final analysis). The results of various treatment modalities used are shown in Table 3. In surgery alone cases, local recurrence was observed in 15.5% and regional nodal metastases developed in 31.05% cases mainly within 1-2 years of follow-up. Both local recurrence rate and regional nodal failure rate were significantly reduced by post operative treatment to the primary site. However, distant metastases pattern was not significantly altered (2.48% *vs* 2.40%). In 83 cases, regional nodes were treated prophylactically by elective inguino-femoral lymph nodal dissection in 28 cases, while 55 cases received prophylactic inguino-femoral radiotherapy of 45 Gy/4 weeks: Using a Cobalt-60 unit. Prophylactic radiotherapy of regional nodes was observed to significantly decrease the regional nodal as well as distant relapse compared to other treatment strategies [Tables 3 and 4]. Follow-up ranged from 2 to 7 years.

**Table 3:** Distribution of cases according to treatment modalities received visa vis loco-regional and distant failure pattern (n = 244)

	Local recurrence (%)	Regional nodal recurrence (%)	Distant metastases (%)
Treatment of primary site			
Surgery alone (n = 161)	25/161 (15.5)	50/161 (31.05)	04/161 (02.48)
Surgery + local radiotherapy (n = 83)	01/83 (01.20)	08/83 (09.63)	02/83 (02.40)
Total = 244	26/244 (10.65)	53/244 (21.72)	06/244 (02.45)
Treatment of regional nodes			
No treatment (n = 91)	10/91 (10.98)	20/91 (21.97)	19/91 (20.87)
Therapeutic radiotherapy (n = 70)	3/70 (04.30)	05/70 (07.14)	08/70 (11.42)
Elective nodal dissection (n = 28)	1/28 (03.57)	04/28 (14.28)	06/28 (21.42)
Prophylactic radiotherapy (n = 55)	2/55 (03.63)	01/55 (01.81)	00/55 (00.00)
Total = 244	16/244 (6.55)	30/244 (12.29)	33/244 (13.52)

**Table 4:** Statistical intercomparison of various follow-up parameters visa vis treatment modalities used

Group comparison	Local recurrence		Nodal recurrence		Distant metastases	
			
	OR	*P* value	OR	*P* value	OR	*P* value
A vs B	15.07	0.000#	04.22	0.00#	01.03	0.971*
C vs D	02.76	0.209*	03.66	0.018#	20.04	0.168*
C vs E	03.33	0.417*	01.69	0.537*	00.97	1.000*
C vs F	03.27	0.209*	15.21	0.002#	29.03	0.001*
D vs E	01.12	1.000*	00.46	0.472*	00.47	0.338*
D vs F	01.19	1.000*	04.15	0.337*	14.19	0.026#
E vs F	00.98	1.000*	09.00	0.077*	30.00	0.002#

(For the primary) – A = 5 Surgery alone; B = Surgery + local radiation (For regional nodes); C = No treatment; D = Therapeutic radiation; E = Elective dissection; F = Prophylactic radiotherapy

* = Not significant

# = Significant; vs = Versus; OR = Odds ratio

## Discussion

In recent years, geographical pathology has gained importance because it throws light on the variations in the incidence of cancer and also helps to unearth and understand the environmental and other ethnic factors that may have a causal association with a particular cancer. Exposure to ultraviolet radiation from sun is the principal cause of skin cancer in most of the countries.[1] Approximately 80% of the ultraviolet induced squamous cell carcinomas develop on the sun-exposed parts of the body like head, face and neck.[3] In contrast, in Kashmir valley, Kangri cancer develops in skin of un-exposed areas of body like thigh, legs and abdominal wall. In this context, our results are in agreement with observations of various other investigators.[10,11] A strong and direct causal relationship exists between the use of Kangri and development of this peculiar skin cancer. Majority of our patients were in the age group of 50-70 years with a male predominance which is in accordance with other studies.[5,6,12] However, Sanyal *et al*. reported higher prevalence among female population. Prolonged use of Kangri induces thermal keratotic changes which take the shape of serpiginous reticular brownish black pigmented lesions [Figures 2 and 3]. Most of these usually resolve spontaneously with discontinuation of Kangri. In the present study all patients had erythema ab igne present for last 7-10 years. The Kangri cancer starts as a nodule and is often associated with itching and occasional bleeding. In more than 70% cases the nodular growth ulcerates with bleeding, discharge and fungation [Figures 4 and 5]. Unlike skin cancers elsewhere in body, Kangri cancer has an aggressive behavior with regional nodal metastases in up to 50% of cases depending upon the T-stage.[1,3,8] The mainstay of treatment in these skin cancer is a timely surgical intervention which is effective and recommended in all cases. Addition of post operative radiotherapy decreases the loco-regional recurrences significantly with odd ratio of 15.07 and 4.22 for local and nodal relapse, respectively. However, distant metastases are not affected significantly in surgery alone vs. Surgery with additional post-operative radiotherapy group [Table 4]. There is dearth of literature regarding the treatment policy to be employed in the patients with clinically negative regional nodes. We have observed a high incidence of regional nodal involvement (more than 30%) in this tumor because of its aggressive behavior and as the ability to identify subclinical nodal disease prior to its manifestation is limited,[3,8,9] prophylactic treatment of the nodes seems justified. It is pertinent to mention here that popliteal nodes are least involved even in tumors of leg situated on antero- lateral/antero-medial aspects. In the present study 28 patients had undergone prophylactic nodal dissection. Of these 4 cases (14.28%) developed nodal recurrence which is lower than what was reported by several other authors.[2,3,8] Fifty five patients received elective(prophylactic) nodal irradiation on a cobalt unit delivering a dose of 45 Gy/4 weeks. In this group, only one (01.8%) patient developed nodal recurrence thereby strongly establishing the role of this approach in eradication of subclinical disease. The statistical intra-group comparison is shown in Table 4.

## Conclusion

Squamous cell carcinoma of skin of extremities and abdominal wall has a peculiar distribution in Kashmir valley as these are directly related to the use of Kangri - an indigenous fire pot used to generate warmth during winter months. Surgery forms the mainstay of treatment in this cancer. This peculiar cancer is very aggressive in clinical behavior with loco-regional recurrence rate of 30-50%. The analysis of various treatment approaches used establishes the fact of benefit of using prophylactic treatment of regional nodes on the pattern of head and neck tumors. In this context, use of prophylactic regional nodal irradiation has proved capable of reducing both the loco-regional and the distant relapse. However, as very little data is available on this aspect, further trials are required to establish its role firmly.
